# The impact of covid-19 on out-of-hours adult hospice care: an online survey

**DOI:** 10.1186/s12904-022-00985-6

**Published:** 2022-06-01

**Authors:** Felicity Hasson, Paul Slater, Anne Fee, Tracey McConnell, Sheila Payne, Dori-Anne Finlay, Sonja McIlfatrick

**Affiliations:** 1 Institute of Nursing and Health Research, School of Nursing, Ulster University, Shore Road, Newtownabbey, BT37 0QB Northern Ireland; 2 Marie Curie Senior Research Fellow, School of Nursing and Midwifery, Queens University Belfast, 97 Lisburn Road, Belfast, BT9 7BL Northern Ireland; 3 International Observatory On End of Life Care, Division of Health Research, Lancaster University, Lancaster, LA1 4YG UK

**Keywords:** COVID-19; pandemic; out-of-hours, Palliative care, Hospice, Survey methodology, Community care

## Abstract

**Background:**

Globally COVID-19 has had a profound impact on the provision of healthcare, including palliative care. However, there is little evidence about the impact of COVID-19 on delivery of out-of-hours specialist palliative care services in the United Kingdom. The aim of the study is to investigate the impact of the COVID-19 pandemic on the delivery of out-of-hours community-based palliative care services.

**Methods:**

A national online census survey of managers of adult hospices in the United Kingdom was undertaken. Survey were emailed to managers of adult hospices (*n* = 150) who provided out-of-hours community palliative care services. Fifteen questions related specifically to the impact of COVID-19. Quantitative data were analysed using descriptive statistics and qualitative data were analysed using descriptive content analysis.

**Results:**

Eighty-one responses to the survey were returned (54% response rate); 59 were complete of which 47 contained COVID-19 data. Findings indicated that COVID-19 impacted on out-of-hours community-based palliative care. To meet increased patient need, hospices reconfigured services; redeployed staff; and introduced new policies and procedures to minimize virus transmission. Lack of integration between charitably and state funded palliative care providers was reported. The interconnected issues of the use and availability of Personal Protective Equipment (*n* = 21) and infection control screening (*n* = 12) resulted in changes in nursing practices due to fear of contagion for patients, carers and staff.

**Conclusions:**

Survey findings suggest that due to increased demand for community palliative care services, hospices had to rapidly adapt and reconfigure services. Even though this response to the pandemic led to some service improvements, in the main, out-of-hours service reconfiguration resulted in challenges for hospices, including workforce issues, and availability of resources such as Personal Protective Equipment. These challenges were exacerbated by lack of integration with wider healthcare services. More research is required to fully understand the implications of such changes on the quality of care provided.

**Supplementary Information:**

The online version contains supplementary material available at 10.1186/s12904-022-00985-6.

## Background

The World Health Organisation (WHO) declared Coronavirus disease (COVID-19) as a global pandemic on 11^th^ March 2020. By May 2021 it has resulted in an estimated 3.3 million deaths worldwide [1], highlighting an increased need for palliative and end-of-life care. The COVID-19 pandemic represents a major challenge to hospice services who have been required to flexibly reconfigure their services in the light of the public health emergency. The term hospice in the United Kingdom refers to predominately independent charitably funded organisations delivering a range of specialist palliative care services including in-patient, out-patient, day care, bereavement support and domiciliary care.

Previous literature has reported that in the pre-pandemic period, hospices were operating with significant challenges (such as high patient acuity, staffing and funding restrictions) [2, 3], however COVID-19 has amplified these issues [4]. A reduced demand for in-patient hospice care and a growing trend for community care has been indicated by recent international studies [5–7]. In a multinational study that aimed to understand challenges faced by palliative care services during COVID-19, authors highlighted an additional workload for service providers (including hospices and community-based care) in order to meet the surge in demand for specialist palliative care [7]. This included: shifting resources (such as moving from inpatient to community provision); educating and upskilling (including supporting people with COVID-19 and support of healthcare staff); and remote working (such as video, and telephone). These measures were accompanied by adherence to national and regional guidelines on reducing the virus transmission which often resulted in internal policy changes to staffing or visiting [8–11]. It has also been reported that since the beginning of the pandemic workforce issues such as staff shortages and managing staff anxiety have posed immense challenges for hospices [7, 10–12]. During the first wave of the pandemic, shortages of Personal Protective Equipment were experienced by 33%-61% of specialist palliative care services in the United Kingdom, and some services also faced shortages of equipment and medication [7, 8].

Although palliative care services are considered to be key in alleviating suffering during a pandemic,[13] very little data exist on how hospices have coped [14], especially in out-of-hours care, or community-based palliative care [5, 15]. Whilst variations in the definition of out-of-hours exist internationally [16], in the United Kingdom, it refers to the period between 18.30–08.00 h and all hours at the weekend and public holiday periods. Given that two thirds of the week are within the out-of-hours period, when unexpected deterioration may occur [17], it has been reported that the provision of out-of-hours care is integral to facilitating community-based palliative care [18, 19]. This study sought to better understand the impact of the pandemic on the delivery of out-of-hours community-based adult palliative care, in order to learn lessons regarding planning and response from COVID-19, to inform future similar crises.

## Methods

### Design

A cross sectional online survey of managers at adult hospices in the United Kingdom was undertaken (Supplementary File 1). This survey was part of a larger national survey that aimed to investigate the United Kingdom hospice healthcare assistant workforce; their role in out-of-hours care provision, and impact of COVID-19. A survey was developed based on a previous survey on COVID-19 and out-of-hours services^.^[6]. The current survey was similar to the previous survey in that it explored palliative and hospice services for palliative care during COVID-19. However, it was different in that the current survey was specific to the United Kingdom and was set within community-based services. The survey was pilot tested with two hospice managers and minor revisions were made based on their feedback. It was also reviewed for face validity by palliative care experts and academics, and reporting adhered to the Checklist for Reporting Results of Internet E-Surveys (CHERRIES) [20], (Supplementary File 2).

### Population/setting

The United Kingdom has 177 hospices, provided by a mixture of independent and National Health Services. Each is set up to respond to local community needs by offering a unique range of services such as inpatient, out-patient clinics, bereavement support, welfare advice and services in the community (in people’s own homes).

### Sample and recruitment

A census of hospices who provided out-of-hours community-based palliative care was used. This was guided by the Hospice UK database, cross referenced with individual hospice websites and confirmed through telephone calls with each hospice to agree eligibility and details of the most appropriate senior person to contact. Eligible hospice managers were invited to participate by email (*n* = 150). Participation was voluntary, with informed consent gained. The survey was simultaneously advertised and promoted on social media (Twitter).

### Data collection

Qualtrics Core XM ™ was used to administer and capture survey responses. The survey and supporting information, along with two reminders (at one and three weeks) were disseminated via email between 5/10/20 and 13/11/20. The overall survey contained 61 questions. Sections were divided into demographics data followed by types of out-of-hours services provided (such as Hospice at Home, Rapid Response, or Advice Line). This was followed by fifteen questions about the impact of COVID-19 on services and workforce. The survey response format comprised a mixture of multi-option tick box (*n* = 39), open text boxes (*n* = 18), and sliding scale (*n* = 4). Where applicable questions included a ‘Don’t Know’ option, and a ‘Back’ button for ease of review. Neither IP addresses nor cookies were used to assign a unique user identifier to each client computer. Time limits for survey completion were not applied. The survey was pilot tested with academics and experts in palliative care (*n* = 5), resulting in minor changes to content (such as inclusion of additional response options).

### Analysis

Data were exported from Qualtrics Core XM ™ to SPSS statistics 25. Following quality assurance of the data, surveys with less than 70% of completed answers were removed. Categorical data were summarized using frequencies and percentages. Percentages were based on the number of respondents answering each question and were rounded. Content analysis was adopted for open-ended questions. For analysis, we gathered qualitative data by content and grouped into four overarching categories [21]. These categories were based on the areas of focus in the main survey. In order to facilitate trustworthiness of the analysis process, original citations were included to illustrate the basis of data categories. Detail on the relationship between data and categories is included in Supplementary File 3.

### Ethical considerations

Information such as the estimated length of time taken to complete the survey, storage of data, and purpose of the study were contained in the participant information sheet (included in the invitation email). Participation in the online survey was voluntary; completion and return of the survey was presumed as informed consent.

## Results

Eighty-one responses to the survey were received (54% response rate). After incomplete forms were removed, 59 responses remained of which 47 contained COVID-19 data. Geographically, these hospices were situated in England (*n* = 39, 79%), Wales (*n* = 3, 9%), Scotland (*n* = 3, 7%) and Northern Ireland (*n* = 2, 5%). The area served by each hospice was described as urban (*n* = 11, 12%), rural (*n* = 3, 7%) or mixed (*n* = 33, 72%); with funding status being a registered charity (*n* = 35, 81%); a registered charity in partnership with NHS (*n* = 10, 16%); or NHS (*n* = 2, 4%). The average number of patient beds were England 17; Wales 12; Scotland 16, and N. Ireland 13. The number of patients seen were England 344 (91); Wales 369 (36.2); Scotland 350 (100) and N. Ireland not recorded. The most common type of out-of-hours service provided by hospices was telephone advice (*n* = 41, 72%), followed by Hospice at Home (*n* = 34, 60%), and Rapid Response (*n* = 20, 35%), with some hospices providing more than one of these options.

Findings of qualitative data were categorized under four main areas: Organisational changes; Patient and Family Carers’ Assessment and Service provision; Staff Impact; Use of Personal Protective Equipment. These areas aim to reflect the focus of the survey which was to shed light on the impact of the COVID-19 pandemic on the delivery of out-of-hours community-based palliative care.

### Organisational changes

Pre-pandemic, hospice providers reported that their level of integration with other community services (such as General Practitioners, or state funded specialist palliative care teams), was ‘very’ or ‘extremely’ integrated (*n* = 18), with one explaining: *‘we wouldn't be able to do our job if we didn't have support from our community teams’* (Hospice 5). By contrast however, a greater number indicated that they were only slightly or somewhat integrated (*n* = 20), and some reported ‘*no integration at all*’ (Hospice 9) (*n* = 5). However, COVID-19 was recognised as heightening demand and placing an additional strain on an already vulnerable community health care system. As the pandemic evolved, nine hospice providers reported a decreased input from general practitioners into their out-of-hours services. Some hospice managers (*n* = 5) reported challenges in gaining access to medication and equipment in out-of-hours periods.

Although for some providers, services remained the same, the majority reported they reconfigured, temporarily suspended, or instigated additional services to respond to demand. For example, one hospice reported they stopped the delivery of respite out-of-hours care, instead “ … *concentrating on symptom management and care of the dying*” (Hospice 48). Other hospices expanded out-of-hours services (*n* = 14) (i.e. hours of availability) or reconfigured existing services, replacing face-to-face visits with telephone or telehealth (i.e. audio-visual, telephone) consultations (*n* = 7). One provider reported that they instigated a new service, integrating their telephone triage in the out-of-hours period and 24/7 rapid response service with two other hospice community teams, enabling the sharing of caseloads, expertise, and staff. There were examples of other services that were extended, or reconfigured:

*“The community team have extended hours until 8 pm and also do an on-call system 8 pm-8am covering the advice line.”* (Hospice 10).

*“More telephone and video contact - this is always first line, except where hands on need … .if still need to visit following this as much to be done on phone/video prior to visit to enable visit to be as short as can be and contact be decreased*” (Hospice 54)

### Patient and family carer’s assessment and service provision

In line with service changes, new procedures and ways of working were also reported. For example, one provider altered their assessment criteria, leading them to introduce a holistic needs assessment for both patients and family carers. This required healthcare professionals to assess patients’ and carers’ needs by telephone. As stated,

*“We have developed a new holistic needs assessment for carers and patients. We have been proactively calling patients to see how they are doing rather than waiting for crisis to hit”* (Hospice 12).

Changes to service delivery were also underpinned by adjustments to internal hospice policies, relating to the provision of care for patients (*n* = 21), and carers (*n* = 20). Policies centred on measures to minimize community transmission of the virus including risk assessments/screening for staff and patients (*n* = 12). For example, in order to identify families who may have come into contact with COVID-19, hospices introduced risk assessments usually by phone before face-to-face visits (*n* = 11). In order to protect staff, some hospices increased screening procedures of staff and patients, and the implementation of government social distancing guidance on the number of people in close proximity, resulted in restrictions on car sharing for hospice staff.

Social distancing measures limited the number of people in the home resulting in a lack of interaction with relatives, which was viewed as a key challenge for hospice staff. Hospices also observed that patients and family members sometimes felt isolated and experienced additional emotional stress due to the pandemic, which had repercussions for staff:

*“Family members are much less socially supported, and this increases burden on healthcare staff to fill the void this creates.”* (Hospice 34)

Evidence also showed that some families were reluctant to have healthcare practitioners in their home, for fear of virus transmission, therefore compounding their sense of isolation.

### Staff impact

Mitigating staff shortages, maintaining safe staffing, and managing the psychological impact of COVID-19 on staff were reported to be amongst their biggest challenges for twenty-one of the hospice providers. Managing staff absence (due to healthcare professional COVID-19 exposure, illness, self-isolation, or the need to care for family members at home) was reported to be challenging. At the time of data collection, respondents (*n* = 27) reported that out-of-hours staff had suspected or confirmed COVID-19. Several respondents (*n* = 11) reported that the pandemic amplified the significant staff pressure which impacted on morale, anxiety, and sickness rates. One hospice believed that anxiety was exacerbated by staff not having access to routine testing (in comparison to other frontline workers). Staff shortages were predominant, with hospices experiencing difficulty around ‘*Capacity to meet need’* (Hospice 27); *‘risk of staff requirement to isolate- reducing pool of staff to deliver services’* (Hospice 41); *‘Maintaining adequate staffing levels, particularly with track and trace affecting attendance’* (Hospice 47) *‘reduced number of staff available’* (Hospice 48).

Efforts to support increased demand, ensure safe staffing, and respond to the risk of staff shortages resulted in hospice providers implementing alternative plans and processes. In addition to changes to working practices, clinical and non-clinical staff (i.e. specialist nurses, healthcare assistants, fundraising and administrative staff) were redeployed into alternative clinical service areas, such as inpatient units and community, and if required, upskilled into that role. As stated,

*“Staff have been redeployed into the service to ensure it could be delivered. services reviewed across all hospice and some adjustments made to reflect day hospice being suspended.”* (Hospice 42)

*“Rapidly inducted other members of the organisation (fundraising team, reception team) into the role of healthcare assistant to fill gaps in the Rota.”* (Hospice 35)

### Use of personal protective equipment

Many hospices reported the introduction of Personal Protective Equipment in community out-of-hours care in response to the pandemic (*n* = 21). One hospice implemented the use of Personal Protective Equipment in situations where COVID-19 was suspected or confirmed, as per government guidance (HSE/NHS England); another reported that all staff wore Personal Protective Equipment ‘regardless’ of the situation; and another described how all staff carried Personal Protective Equipment, and wore it if they were uncertain or if there were visitors that were from outside the country. Although the use of Personal Protective Equipment was described as being key in the pandemic response (*n* = 12), hospices cited challenges such as insufficient or inadequate Personal Protective Equipment (*n* = 6) or barriers posed by the use of Personal Protective Equipment (*n* = 17). For example, therapeutic touch and facial expressions used to convey compassion were believed to be negatively affected by social distancing and Personal Protective Equipment. Specifically, respondents reported that wearing Personal Protective Equipment was a barrier to proving end-of-life care, resulting in a lack of interaction with relatives and/ or patient. As stated,

*“Not touching a family member when all they want is human comfort in one of the times when they are most vulnerable and need comfort after someone has died has been the hardest thing. Watching someone in distress causes distress to the nurse/Healthcare Assistant too”* (Hospice 17)

*“Wearing/ use of PPE can cause difficulties for the team supporting loved ones especially if they are of an older generation. It poses barrier to communication and completely takes away the use of therapeutic touch often used to reassure people”.* (Hospice 24)

## Discussion

Survey findings suggest that the COVID-19 pandemic resulted in community-based palliative care services rapidly responding and adapting already stretched services in response to increased patient need. Similar to findings from Italy [8], United Kingdom hospices experienced unprecedented workforce and service-related challenges, which were addressed by re-configuring services, re-deploying staff and adhering to strict virus control measures. However, these newly implemented practices were often instituted against a backdrop of a shortage of resources, lack of integration with wider healthcare services (such as general practitioners), and lack of standardized guidance to ensure optimal care for patients or support for staff.

In our survey, hospice managers highlighted families’ reluctance to have healthcare practitioners in their home for fear of virus transmission. However, findings also revealed a reluctance by families to attend in-patient hospice facilities for the same reason. For some, this posed tension around decisions about ongoing care. This situation was exacerbated by adherence to COVID-19 related restrictions (such as social distancing), and potentially impacted on the provision of end-of-life care. Given the vulnerability of palliative care patients and families in a community setting, the provision of compassionate care, including therapeutic touch and clear communication is essential. Findings from a recent study, suggest that an inability to provide such therapeutic care may lead to negative psychosocial effects for the patient and family carer [11]. Similarly, in Singapore, a study that examined the impact of adherence to national guidelines for social distancing for hospice home care staff, findings revealed that such restrictions (including limiting the number of family members during end-of-life care) often resulted in angry and distressed family members [22].

Reflective of research from the United States [10], our survey findings also highlighted concerns about staff wellbeing, suggesting that fears about providing palliative care within COVID-19 related restrictions resulted in elevated stress levels amongst the hospice and palliative care workforce. According to several managers who responded to our survey, staff anxiety among the hospice workforce was high, and was compounded by the number of staff who had tested positive for the virus; absence of testing; and lack of, or inadequate Personal Protective Equipment. Other recent studies have highlighted that inadequate Personal Protective Equipment for healthcare workers, lack of routine testing and lack of consideration of existing skills of redeployed staff contributed to workforce problems [12, 21].

It has previously been argued that staff shortages and lack of Personal Protective Equipment in the United Kingdom were more common among charity-funded than public healthcare services, and that community palliative care should be fully integrated with the national health system in order to access resources to meet the COVID-19 surge in patient need [6]. However, our survey findings suggested that the majority of respondents were charitably funded hospice providers, with limited integration with other healthcare services, which is concerning, given the World Health Assembly [23] endorsement of integration of palliative care services for optimum patient care. Although some evidence in our survey indicated sharing of resources (such as one hospice joining with another for their rapid response service), this practice appeared to be ad hoc and does not go far enough in embedding a culture of partnership between charitably and state funded hospice providers that could improve patient outcomes. For this to occur, charitably managed services need to be recognised as equal to state funded services (such as National Health Service), and to be resourced on this basis. Such a partnership could potentially result in improvements to sharing of information, standardisation of procedures or collaboration around targeting of resources (such as medication or Personal Protective Equipment). However, given that this is the view of hospice managers, it would be important to have the views of relatives, and other hospice staff in order to better understand the extent of the impact of COVID-19 on out-of-hours community-based palliative care. Therefore further work in this area is warranted.

Despite the negative consequences of rapid change for community based out-of-hours palliative care services brought about by COVID-19, some evidence in our study also indicates that as a result of these changes, certain services were improved, according to hospice managers. For example, one hospice developed a new ‘proactive’ needs assessment, and another restructured their hospice at home service resulting in an extended service. Embedding such improvements in practice over the longer term and learning from the COVID-19 response will be key as community-based healthcare providers move forward [24].

### Strengths and limitations

A key strength of this study is that it was United Kingdom wide and as such reflects the response to the pandemic that includes all nations in the United Kingdom. However, the relatively low response rate means that some important data about the impact of the pandemic on community-based out-of-hours service could have been missed. That being said, in light of the extreme pressure on hospice services during the pandemic it is not surprising that the response rate was low. Furthermore, the cross-sectional nature of the survey meant that data was a ‘snapshot’ in autumn 2020. Rapidly changing circumstances and practices potentially meant that data captured at one point in time may not have been truly reflective of the extent of the impact of the pandemic on service delivery. Finally, only the views of managers were sought for the survey. As the demographic background of the respondent managers was not obtained it cannot be determined whether or not they had a clinical background (which may have influenced how they interpreted survey questions), which is seen as a limitation. Also, the inclusion of managers to respond to the survey means that the perspectives of others, such as patients and families is missing. The views of patients and families about the impact of COVID-19 within out-of-hours community palliative care remains an important area for future research.

## Conclusion

The aim of our survey was to better understand the impact of the COVID-19 pandemic on the delivery of out-of-hours community-based palliative care, in order to learn lessons regarding planning and response in such crisis situations. Findings suggested that a surge in patient need for community-based out-of-hours resulted from a reluctance of patients and families to attend in-patient facilities due to fear of infection. The surge in patient need for community based palliative care led to hospice providers rapidly reconfiguring community services. This added additional pressure to already stretched services and exacerbated an existing lack of integration between hospice providers and wider healthcare services. Even though this response to the pandemic led to some service improvements, in the main, COVID-19 related service reconfiguration resulted in challenges for hospices, including workforce issues, and availability of resources such as Personal Protective Equipment. The impact of these changes on the quality of care delivered from the patient and carer perspective is currently unknown. Findings of this study suggest a need for further research on how we improve preparedness and integration among specialist palliative care services. This may improve access to resources such as medication during the out-of-hours period or Personal Protective Equipment, potentially resulting in better patient and family outcomes, and enabling more people to die at home if desired.

## Supplementary Information

**Additional file 1.**

**Additional file 2.**

**Additional file 3.**

## Data Availability

The dataset supporting the conclusions of this article is available on request from the corresponding author.

## Abbreviations

CHERRIES: Checklist for Reporting Results of Internet E-Surveys
UK: United Kingdom
