# Supplementary material for: The impact of covid-19 on out-of-hours adult hospice care: an online survey
Source: BMC Palliat Care. 2022 Jun 1;21:94. doi: 10.1186/s12904-022-00985-6 (PMC9155980; doi:10.1186/s12904-022-00985-6)
Supplement: Supplementary file 3 — Additional file 3. [file 12904_2022_985_MOESM3_ESM.docx]

**Supplementary File 3**

**Categorisation of data**

| **Example of data** | **Sub-Category** | **Generic Category** | **Category** |
| --- | --- | --- | --- |
| ‘Availability of other services are reduced which impacts on the demand for our service’ (Hospice 23)  Key factor in delivering OOH care during COVID: ‘Timeliness of getting to patient because the GP is not seeing the patients as they would have previously (Hospice 15)  ‘GPs were not providing many face to face visits.  DNs continued to visit and to contact the hospice for advice and support with patients who were  symptomatic/dying’ (Hospice 41) | External service provision | Levels of integration | Organisational changes |
| ‘The community team have extended hours until 8pm, and also do an on-call system 8am-8pm covering the advice line. (Hospice 10) | Reconfiguration of services | Responding to rapidly changing circumstances |  |
| “…concentrating on symptom management and care of the dying” (Hospice 48). | Prioritisation |  |  |
| “We have developed a new holistic needs assessment for carers and patients. We have been proactively calling patients to see how they are doing rather than waiting for crisis to hit” (Hospice 12).  ‘Introduced a telephone risk assessment so attempt to identify patients and families that may have come into contact with COVID 19’ (Hospice 9)  ‘There are COVI-19 questionnaires that must be filled out on every visit. If a patient is suspected covid we are unable to visit them’ (Hospice 24) | Risk assessment for families | Assessment | Patient and family carers assessment and service provision |
| (Barrier) ‘Being able to reach our patients when they are not comfortable with technology’ (Hospice 18) | Reduction in face-to-face consultations/increase in video technology | Isolation |  |
| ‘Family members are much less socially supported and this increases burden on healthcare staff to fill the void this creates’ (Hospice 34)  ‘Lack of touch due to social distancing, making it difficult to support those who are distressed’ (Hospice 35) | Impact of social distancing measures on care |  |  |
| A Key Challenge: ‘Managing staff fear and anxiety throughout the pandemic’ (Hospice 9)  A Key challenge: Families and patients being fearful of COVID. Staff being fearful of COVID (Hospice 5) | Fear and anxiety | Psychological impact on staff | Staff Impact |
| ‘A small number of staff shielded so there was less availability as we faced increased demand’ (Hospice 45)  ‘Maintaining adequate staffing levels, particularly with track and trace affecting attendance’ (Hospice 47)  ‘There is now an element of fatigue amongst the staff as they are unable to congregate with families and friends out of hours to support their own resilience’ (Hospice 37) | Staff absence due to sickness/fatigue and isolating | Changes in staffing levels |  |
| ‘Rapidly inducted other members of the organisation (fundraising team, reception team) into the role of Healthcare assistant to fill gaps in the rota’ (Hospice 35)  ‘Reduced number of IPU beds and have moved services to the community. More HCA's working in community’ (Hospice 48) | Staff up-skilling | Changes to workforce practices |  |
| ‘Wearing/use of PPE can cause difficulties for the team supporting loved ones especially if they are of an older generation. It poses a barrier to communication and completely takes away the therapeutic touch often used to reassure people’ (Hospice 24) | Barrier to therapeutic support | Increased use of PPE | Use of PPE |
| A key factor in delivering OOH care in the pandemic: ‘Having enough PPE and infection control protocols to ensure staff and patient and family safety’ (Hospice 18) | Logistical concerns (consistency and availability) of PPE |  |  |
